# Bacterial Community Responses to Soils along a Latitudinal and Vegetation Gradient on the Loess Plateau, China

**DOI:** 10.1371/journal.pone.0152894

**Published:** 2016-04-05

**Authors:** Quanchao Zeng, Yanghong Dong, Shaoshan An

**Affiliations:** 1 College of Natural Resources and Environment, Northwest A&F University, Yangling, Shaanxi, P.R. China; 2 State Key Laboratory of Soil Erosion and Dry Land Farming on Loess Plateau, Northwest A&F University, Yangling, Shaanxi, P.R. China; Loyola University Chicago, UNITED STATES

## Abstract

Soil bacterial communities play an important role in nutrient recycling and storage in terrestrial ecosystems. Loess soils are one of the most important soil resources for maintaining the stability of vegetation ecosystems and are mainly distributed in northwest China. Estimating the distributions and affecting factors of soil bacterial communities associated with various types of vegetation will inform our understanding of the effect of vegetation restoration and climate change on these processes. In this study, we collected soil samples from 15 sites from north to south on the Loess Plateau of China that represent different ecosystem types and analyzed the distributions of soil bacterial communities by high-throughput 454 pyrosequencing. The results showed that the 142444 sequences were grouped into 36816 operational taxonomic units (OTUs) based on 97% similarity. The results of the analysis showed that the dominant taxonomic phyla observed in all samples were *Actinobacteria*, *Proteobacteria*, *Chloroflexi*, *Acidobacteria* and *Planctomycetes*. *Actinobacteria* and *Proteobacteria* were the two most abundant groups in all samples. The relative abundance of *Actinobacteria* increased from 14.73% to 40.22% as the ecosystem changed from forest to sandy, while the relative abundance of *Proteobacteria* decreased from 35.35% to 21.40%. *Actinobacteria* and *Proteobacteria* had significant correlations with mean annual precipitation (MAP), pH, and soil moisture and nutrients. MAP was significantly correlated with soil chemical and physical properties. The relative abundance of *Actinobacteria*, *Proteobacteria* and *Planctomycetes* correlated significantly with MAP, suggesting that MAP was a key factor that affected the soil bacterial community composition. However, along with the MAP gradient, *Chloroflexi*, *Bacteroidetes* and *Cyanobacteria* had narrow ranges that did not significantly vary with the soil and environmental factors. Overall, we conclude that the edaphic properties and/or vegetation types are driving bacterial community composition. MAP was a key factor that affects the composition of the soil bacteria on the Loess Plateau of China.

## Introduction

The Loess Plateau in China is one of the most eroded areas in the world. Accordingly, the “Grain-for-Green Program” was implemented on a large scale by the central government beginning in 1999 in which vegetation restoration was implemented in this area to remediate the soil degradation problem. Bacteria are a dominant group of soil organisms [1] and play an essential role in ecosystem recovery [2]. Over the past few decades, an increasing number of researchers have been investigating the bacterial diversity in different soils, particularly during the process of vegetation restoration. Bacteria have strong effects on soil processes, and management and land uses have effects on the soil bacterial diversity [3]. However, there is little information regarding the long-term effects of vegetation restoration on the soil bacterial communities on the Loess Plateau in China. Vegetation types have large direct or indirect effects on soil community composition and diversity. The effects of alterations in the vegetation types on the soil physical and chemical properties have been well-studied. Due to the changes in plant species composition, vegetation types can have significant and long-lasting effects on soil carbon and nutrient contents, soil texture, and pH [4–6]. The effects of the vegetation types and soil properties affect the groups of soil organisms [7].

Previous studies have focused exclusively on plant and animal taxa, showing that macroorganisms such as trees and animals exhibit biogeographical distributions [8]. However, it is not known whether the microorganisms such as fungi and bacteria vary along a latitudinal gradient. Stegan et al. [9] found that the microbial species were widely distributed and that the microbial community composition was governed by ecological Drift and Selection. Fierer et al. [10] suggested that geographic distance should be the best predictor of genetic divergence among communities. Liu et al. [11] used a high-throughput sequencing method to explore the diversity in the black soils in northeast China. The results suggested that a latitudinal diversity gradient of the bacterial communities might be present in the black agriculture soil zone. So far, similar studies on a large scale in disturbed ecosystems in the Loess Plateau are still limited.

Soil is a complex environment, within which the types of microorganisms are associated with the soil properties, latitude, vegetation types, and other factors. Therefore, understanding the diversity of soil microorganisms along a latitudinal gradient has important ecological significance. The development of high-throughput sequencing technologies such as 454 pyrosequencing currently offers an opportunity to effectively sequence DNA fragments, which substantially improves the researcher’s ability to detect non-dominant microbial communities. Next-generation sequencing technologies have made high-throughput sequencing easy and inexpensive to implement [12].

In this study, we selected five vegetation zones (forest, forest-grass, grass, sand and desert ecosystems) as subjects, which represented the vegetation ecosystems from south to north in the Loess Plateau. Our objectives were to determine the effects of edaphic properties and soil properties on the diversity of soil microbial communities using high-throughput 454 pyrosequencing technology.

Our objectives for this study were (i) to determine the abundance, taxonomic diversity and composition of the bacterial communities across the 600 km geodistance of the Loess Plateau in Shaanxi province; (ii) to directly compare the variability in the bacterial communities across this 600 km geodistance with the variability among soils collected from the wide range of mean annual precipitation from North to South; and (iii) to determine which factors most strongly affect the soil bacterial communities.

## Materials and Methods

### Experimental design and soil sampling

This study was performed in Yulin City, Jingbian City, Liandaowan Citiy, Ansai City and Fuxian City in the northern Loess Plateau (108°41′40.27′′-109°58′10′′E, 36°03′37.16′′-38°55′9.63′′N), China. These areas repented five vegetation stages after the implementation of “Grain-for-Green Program”. The study areas are characterized by a semi-arid climate and a deeply incised hilly-gully Loess landscape. The slopes are between 0° and 20°. Sites stabilized through vegetation restoration for different vegetation ecosystems provide an ideal opportunity to understand vegetation types in extreme environments.

A sandy ecosystem is located in the city of Jingbian, and a desert ecosystem is located in the city of Yulin. These two regions have similar dominant plant species, including *Artemisia ordosica*, *Hedysarum scoparium* and *Hedysarum leave* and other herb species. The soils in the two ecosystem are sand soil with less vegetation, and the soils in the forest, forest-grass and grass ecosystems are loess-derived. In the forest ecosystem, *Platycladus orientalis*, *Quercus wutaishanica* and *Robinia pseudoacacia* predominate. The grass ecosystem is dominated by *Artemisia giraldii*, *Artemisia vestita* and *Stipa bungeana*. However, the forest-grass ecosystem has trees, shrubs and grasses, including *Robinia pseudoacacia*, *Artemisia vestita* and *Sophora moorcroftiana*.

Along the latitudinal gradient from 36° to 39°, the mean annual temperature (MAT) decreases from 11°C to 8°C and the mean annual precipitation (MAP) decreases from 560 mm to 390 mm. From south to north, there were five types of vegetation ecosystems (forest, forest-grass, grass, sandy and desert ecosystems). The field sites do not contain endangered or protected species and did not require research permission.

**Fig 1:**
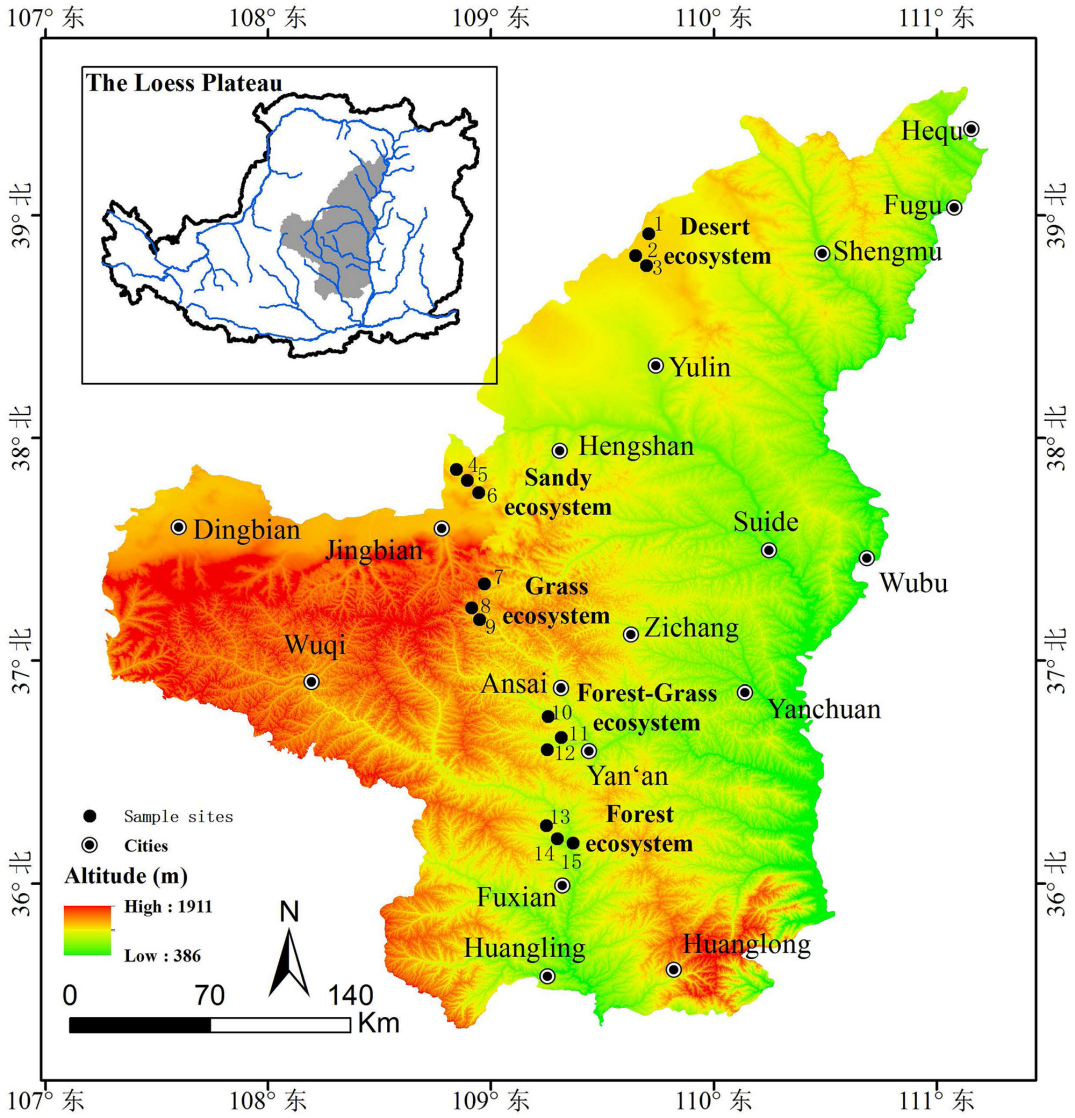
The descriptions of soil sample sites.

In August 2013, when the vegetation community biomass had peaked, 15 sites were selected on the basis of five vegetation ecosystems (forest, forest-grass, grass, sandy and desert ecosystems). These sites were separated by 2 to 2.5 km (Table 1 and Fig 1). At each site, we set up five plots of 20 m×20 m. Plots in both sites were at least 1000 m apart. In each plot, five quadrants (1 m×1 m) were individually chosen, one in each of the four corners and one in the center of the plot. Soil samples were taken at five points in the quadrants of each plot, and soil samples in 0–10 cm soil layer were collected. After carefully removing the surface organic materials and fine roots, each mixed soil sample was divided into two parts. One part of the soil sample was air-dried for the evaluation of the soil properties. The other part was frozen at -80°C (liquid nitrogen) for subsequent high-throughput 454 pyrosequencing analysis. The collected samples were analyzed for physicochemical properties or used for DNA extraction.

**Table 1:** The descriptions of soil sampling sites.

Ecosystem type	Locations	MAT(°C)	MAP(mm)	Longitude (°)	Latitude (°)	Altitude (m)	Dominant plant species
				109.75	38.92	1192	*Artemisia ordosica*, *Hedysarum scoparium*, *Hedysarum laeve*
Desert	Yulin	8.7	390.3	109.65	38.82	1192	*Artemisia ordosica*, *Hedysarum scoparium*, *Hedysarum laeve*
				109.7	38.38	1120	*Artemisia ordosica*, *Hedysarum scoparium*, *Hedysarum laeve*
				108.75	37.65	1333	*Artemisia ordosica*, *Hedysarum scoparium*, *Hedysarum laeve*
Sandy	Jingbian	9.4	412.6	108.69	37.64	1349	*Artemisia ordosica*, *Hedysarum scoparium*, *Hedysarum laeve*
				109.08	37.86	1229	*Artemisia ordosica*, *Hedysarum scoparium*, *Hedysarum laeve*
				108.97	37.24	1463	*Artemisia giraldii*, *Artemisia vestita*
Grass	Liandaowan	9.1	499.2	108.99	37.18	1341	*Artemisia giraldii*, *Artemisia vestitaa*
				108.97	37.25	1406	*Artemisia giraldii*, *Artemisia vestita*
				109.25	36.75	1166	*Sophora moorcroftiana*, *Artemisia giraldii*
Forest-Grass	Ansai	10.3	501.8	109.26	36.75	1153	*Robinia pseudoacacia*, *Artemisia giraldii*
				109.31	36.86	1192	*Artemisia vestita*, *Artemisia giraldii*
				109.15	36.06	1333	*Platycladus orientalis*, *Artemisia giraldii*
Forest	Fuxian	9.5	518.3	109.15	36.06	1327	*Quercus wutaishanica*, *Artemisia giraldii*
				109.17	36.08	1193	*Robinia pseudoacacia*, *Artemisia giraldii*

### Selected soil properties

The soil pH was determined on the air-dried samples (sieved to 1 mm) using a glass electrode meter in a suspension of 1:2.5 soil/water ratio (w/v). Soil moisture was determined gravimetrically in fresh soils at 105°C overnight, and the water content was expressed as a percentage of the dry weight. The soil bulk density (BD) was determined using the auger-hole method [13]. The fumigation-extraction method was used to determine the soil microbial biomass (MBC) [14]. The organic matter (SOM) content was analyzed using dichromate oxidation [15]. Total N (TN) was measured using the Kjeldahl method [16]. Total phosphorus (TP) was measured spectrophotometrically after wet digestion with H_2_SO_4_ and HClO_4_ [17]. Available P (AP) was extracted using the Olsen bicarbonate method. NH_4_^+^-N (AN) and NO_3_^−^-N (NN) were measured using a Seal AutoAnalyzer3.

### DNA extraction and sequencing PCR

Soil DNA was extracted from 0.5 g of the mixed soil samples using a Soil DNA kit (OMEGA, Bio-Tek, GA, USA) according to the manufacturer’s instructions. An aliquot of the extracted DNA from each sample was used as a template for amplification. The V1-V3 hypervariable regions of the bacterial 16S rRNA gene were amplified with primers 27F (5′-AGAGTTTGATCCTGGCTCAG-3′) and 533R (5′-TTACCGCGGCTGCTGGCAC-3′) containing the A and B sequencing adapters (454 Life Sciences) [11].

The 20 μl PCR reaction system contained 4 μl 5× FastPfu Buffer, 2.5 μl mM dNTPs, 0.8 μl forward and reverse primers (5 μM), 0.4 μl FastPfu polymerase (TransStart Fastpfu DNA Polymerase, TransGen) and 10 ng of the template DNA. The PCR amplification was performed using an ABI GeneAmp 9700 instrument. The PCR conditions used for the amplification of the 16S rRNA gene were as follows: 2 min at 95°C; 25 cycles of 30 seconds at 95°C for denaturation, 30 seconds at 55°C for annealing and 30 seconds at 72°C for extension; and 5 min at 72°C. For each sample, 20 parallel PCRs (including two negative control reactions) were performed, and the PCR products were evaluated by analyzing 2 μl of the product on a 2% agarose gel. Next, all of the PCR products from the same sample were pooled, and PCR products of an approximate size of 400 bp were purified using a PCR Purification kit (Axygen Bio, USA). After purification and quantification using QuantiFluor^™^-ST (Promega, U.S.), a mixture of the amplified products was used for pyrosequencing on a Roche 454 GS FLX+ Titanium platform (Roche 454 Life Sciences, Branford, CT, U.S.) according to standard protocols [18]. Pyrosequencing was carried out on a 454 Life Sciences Genome Sequence FLX (Roche) at the Shanghai Majorbio BioPharm Technology Co., Ltd., Shanghai, China.

### Statistical and bioinformatics analysis

Several statistical analyses were performed separately on the soil property datasets using the statistical package for the social sciences (SPSS version 20.0 for Windows), including one-way ANOVA and S-K-N multiple range comparison (P = 0.05). The relationships between soil bacterial composition and the environmental factors were tested using linear regression analyses using SPSS 20.0 for Windows. The relationships between the soil bacterial composition and properties were evaluated using R.

The resulting sequences were processed using Mothur software [19]. Briefly, the raw reads were first assigned to samples according to their tags and then the standard primers and barcodes were trimmed off, after which reads with length less than 200 bp or with ambiguous characters were removed. The chimeric sequences were also excluded by the chimera.uchime command with default parameters. After removing the barcode and primer sequences, the unique sequences were aligned against the reference sequence database (Silva database). The remaining reads were pre-clustered (http://www.mothur.org/wiki/Pre.cluster) and then clustered using uncorrected pairwise algorithm. In addition, Operational taxonomic units (OTUs) were defined as sharing >97% sequence identity using Furthest neighbor method (http://www.mothur.org/wiki/Cluster). An OTU-based analysis was performed to calculate the richness and diversity indices (Ace, Coverage, Chao, Simpson and Shannon) with a cutoff of 3% dissimilarity. Raw sequence data in FASTQ format are accessible from the NCBI SRA study number SRP070625, accession numbers SRX1602057- SRX16072.

## Results

### Effects of MAP on soil biogeochemical properties

The ecosystem types had significantly different soil properties (Table 2). The soil pH and BD varied from 7.95 to 8.36 and from 0.94 to 1.66 g/cm^3^, respectively. The soil pH and BD were the lowest in the forest ecosystem and highest in the desert ecosystem. SOM content of the soils differed as a function of the vegetation types. The concentration of SOM in the forest ecosystem were significantly higher than the other ecosystems. TN content in samples from the forest ecosystem was the highest, whereas this value was lowest in the sandy ecosystem. TP content was highest in the forest and forest-grass ecosystems and lowest in the sandy ecosystem. The ratio of C/N in the desert ecosystem was significant higher than that in grass and forest ecosystem, and the ratio of N/P in the forest and forest-grass ecosystems was significant higher than that in other ecosystems. MBC ranged from 552.05 mg/kg in the forest ecosystem to 20.18 mg/kg in the sandy ecosystem, and it consistently decreased as MAP increased. Overall, the forest ecosystem with higher MAP had the highest level of nutrients, while sandy and desert ecosystems had the lowest. Overall, from south to north, along the increasing MAP gradient, soil pH and BD increased and SM, SOM, TN, TP, NN, AN, AP, AK and MBC decreased.

**Table 2:** Biogeochemical properties of soils under different vegetation types.

Vegetation ecosystems	Forest	Forest-grass	Grass	Sandy	Desert
pH	7.95±0.12c	8.13±0.05b	8.22±0.03ab	8.33±0.06a	8.36±0.14a
BD (g/cm^3^)	0.94±0.05c	1.14±0.06bc	1.19±0.04b	1.50±0.10a	1.66±0.05a
SM	0.18±0.04a	0.14±0.03a	0.06±0.01b	0.02±0.02b	0.01±0.01b
SOM (g/kg)	37.66±16.93a	15.76±5.83b	8.41±0.49b	2.14±1.23b	3.62±1.21b
TN (g/kg)	1.91±0.72a	1.14±0.52ab	0.51±0.06bc	0.09±0.03c	0.13±0.04c
TP (g/kg)	0.57±0.04a	0.55±0.01a	0.47±0.02b	0.15±0.03c	0.22±0.05c
NN (mg/kg)	5.11±3.26bc	9.34±2.75a	6.13±1.57ab	3.21±0.14bc	1.50±0.58c
AN (mg/kg)	9.33±2.78a	7.81±2.67a	6.01±1.30a	5.84±3.63a	3.42±0.77a
AP (mg/kg)	2.27±0.17a	2.58±0.27a	1.76±0.24b	1.17±0.14c	2.42±0.26a
AK (mg/kg)	530.45±25.02a	328.88±64.74b	325.09±39.70b	173.56±14.70c	209.99±26.42bc
MBC (mg/kg)	552.05±178.91a	310.05±105.37b	161.27±60.2bc	20.18±6.43c	22.64±3.04c
C/N	11.14±1.62b	8.33±0.87b	9.59±0.73b	12.81±3.83ab	16.07±2.48a
C/P	38.58±17.32a	16.67±5.97b	10.28±0.34b	7.75±3.21b	9.51±1.19b
N/P	3.36±1.20a	2.08±0.92ab	1.08±0.1bc	0.59±0.08c	0.60±0.04c

Soil bulk density, BD; Soil organic matter, SOM; SM, Soil moisture; TN, Total nitrogen; TP, Total phosphorus; NN, Nitrate nitrogen; AN, Ammonium nitrogen; AP, Available phosphorus; AK, Available potassium; MBC, Soil microbial biomass carbon; C/N, The ratio of total organic carbon to total nitrogen; C/P, The ratio of total organic carbon to total phosphorus; N/P, The ratio of total nitrogen to total phosphorus. Different lowercase letters showed significant differences under different vegetation ecosystems in the same phyla (P < 0.05).

### Bacterial community composition

Across all of the soil samples, we obtained a total of 36816 OTUs with 1593–2829 OTUs per sample (mean 2454). In the grass ecosystem, Shannon’s diversity index, Chao index and Ace index were highest, and the Simpson diversity index was lowest. In contrast, the Simpson diversity index was the highest in the sandy ecosystem (Table 3). Vegetation ecosystems had no significant effect on Shannon’s diversity index, Chao index and Ace index (P>0.05).

**Table 3:** Characteristics of soil bacteria richness and diversity indices under different vegetation types (Means ± SD, n = 3).

Vegetation systems	Reads	0.97					
		OTU	Ace	Chao	Coverage	Shannon	Simpson
Desert	9462±1167a	2392±186a	3326±271a	3184±237a	0.90±0.01a	7.12±0.07a	0.0018±0.0005a
Sandy	9364±1641a	2243±578a	3204±868a	3123±829a	0.91±0.02a	6.90±0.42a	0.0031±0.0021a
Grass	10062±604a	2810±27a	4033±114a	3909±112a	0.89±0.01a	7.31±0.03a	0.0014±0.0001a
Forest-grass	9190±1680a	2589±306a	3863±494a	3709±469a	0.88±0.02a	7.21±0.10a	0.0015±0.0002a
Forest	9404±956a	2238±233a	3194±404a	3138±350a	0.90±0.01a	6.90±0.13a	0.0028±0.0008a

Different lowercase letters showed significant differences under different vegetation ecosystems in the same phyla (P < 0.05).

**Fig 2:**
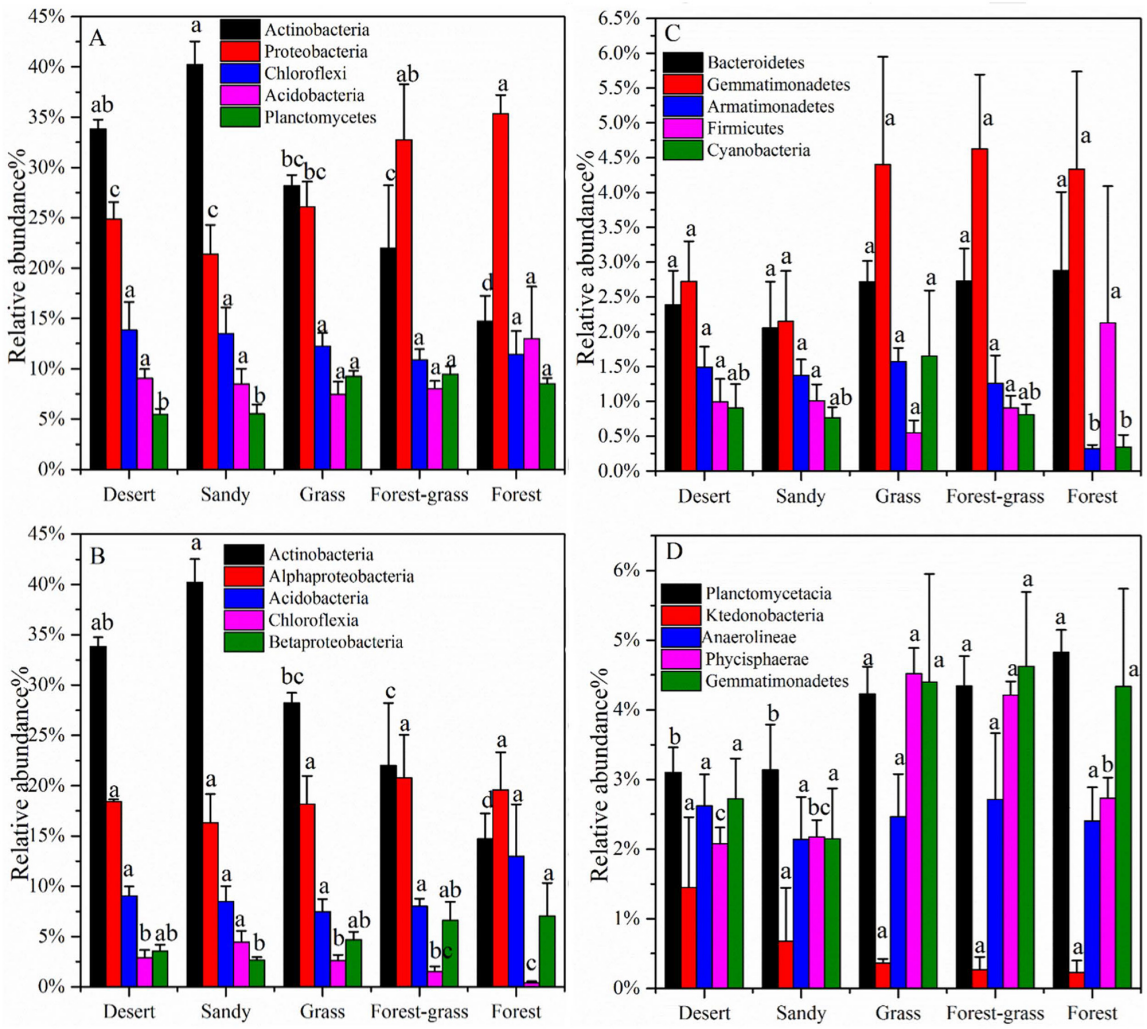
Relative abundance in the bacterial community composition at the phylum level (A and C) and at the class level (B and D) within the different ecosystems. Values designated by different lowercase letters showed significant differences under different vegetation ecosystems in the same phyla, the same as followings (*P* < 0.05).

As shown in Fig 2A, at the phylum level, the structures of the microbial communities differed in terms of both the predominant phylum and the relative abundance of each phylum. *Actinobacteria* (12.66–42.96%), *Proteobacteria* (17.45–40.33%), *Chloroflexi* (9.25–16.25%), *Acidobacteria* (7.10–20.21%), *Planctomycetes* (4.70–10.01%), *Bacteroidetes* (1.52–4.47%), *Gemmatimonadetes* (1.17–6.29%), *Armatimonadetes* (1.17–6.29%) and *Cyanobacteria* (0.32–2.98%) were the 9 most abundant phyla. In all samples, *Actinobacteria* and *Proteobacteria* were the most abundant phyla, which together accounted for 46.89–67.23% of all bacterial sequences obtained from all of the soil samples. The relative abundance of *Actinobacteria* was much higher in the sandy ecosystem than in other ecosystems. *Proteobacteria* were the second highest bacterial abundance found in all samples. The relative abundance of these bacteria was highest (35.35%) in the forest ecosystem and lowest in the sandy ecosystem (21.40%). The ANOVA test indicated that *Actinobacteria*, *Proteobacteria* and *Planctomycetes* differed significantly under different ecosystems (P<0.05) and that *Planctomycetes* were significantly more abundant in forest and grass ecosystem soils (P<0.05). Compared with the *Actinobacteria* and *Proteobacteria*, *Chloroflexi*, *Plantctomycetes* had narrow ranges, which accounted for 10.90–13.86%, 2.05–2.88% and 0.34–1.65%, respectively (Fig 2C). And for the three phyla, there was no significant differences between different vegetation ecosystems (P>0.05).

At the class level, *Actinobacteria*, *Alphaproteobacteria*, *Acidobacteria*, *Chloroflexia* and *Betaproteobacteria* were dominant communities. Vegetation ecosystems had significant effects on soil bacterial communities (P<0.05). The relative abundance of *Actinobacteria* for sandy ecosystem was significant higher than grass, forest-grass and forest systems (Fig 2B). No significant differences for *Alphaprotebacteria*, *Acidobacteria*, *Ktedonobacteria*, *Anaerolineae* and *Gemmatimonadetes* were detected in different ecosystems.

Of all of the edaphic and climate variables examined, only altitude and MAT had no significant association with soil bacterial communities (Table 4). There was a significant negative correlation of *Actinobacteria* with SM, MAP, SOM, TN, TP, NN, AN, AP, AK, MBC, C/P and N/P and a significant positive correlation between pH and BD. However, *Proteobacteria* had the opposite correlations with the edaphic and climate variables. Meanwhile, there were no significant correlations between *Chloroflex*, *Bacteroidetes* and *Cyanobacteria* and edaphic and climate variables (Table 4).

**Table 4:** The Pearson correlations between the relative abundances of the 9 dominant bacterial phyla and environment factors.

	Actinobacteria	Proteobacteria	Chloroflexi	Acidobacteria	Planctomycetes	Bacteroidetes	Gemmatimonadetes	Armatimonadetes	Cyanobacteria
MAP	-0.817**	0.666**	-0.456	0.247	0.890**	0.291	0.651**	-0.467	-0.088
MAT	-0.433	0.484	-0.379	0.011	0.533*	0.121	0.336	-0.255	-0.224
Altitude	0.175	-0.184	-0.241	0.023	0.023	0.176	-0.101	0.036	0.401
pH	0.794**	-0.733**	0.515*	-0.553*	-0.567*	-0.307	-0.387	0.640**	0.384
BD	0.781**	-0.611*	0.444	-0.516*	-0.770**	-0.391	-0.463	0.574*	0.252
SM	-0.916**	0.804**	-0.430	0.580*	0.668**	0.366	0.535*	-0.748**	-0.460
SOM	-0.800**	0.769**	-0.470	0.631**	0.451	0.274	0.288	-0.768**	-0.395
TN	-0.869**	0.862**	-0.503*	0.523*	0.562*	0.231	0.413	-0.754**	-0.395
TP	-0.871**	0.747**	-0.381	0.164	0.889**	0.218	0.672**	-0.463	-0.105
NO_3_-N	-0.583*	0.551*	-0.244	-0.113	0.676**	0.302	0.619*	-0.175	-0.147
NH_4_-N	-0.562*	0.656**	-0.183	0.196	0.442	0.084	0.063	-0.616*	-0.182
AP	-0.620*	0.644**	-0.140	0.04	0.372	0.177	0.365	-0.200	-0.167
AK	-0.796**	0.702**	-0.418	0.412	0.533*	0.500*	0.459	-.648**	-0.318
MBC	-0.893**	0.835**	-0.46	0.598*	0.609*	0.372	0.362	-.740**	-0.377
C/N	0.410	-0.360	0.459	0.020	-0.634**	-0.184	-0.553*	0.127	0.098
C/P	-0.752**	0.712**	-0.424	0.716**	0.356	0.300	0.211	-.772**	-0.432
N/P	-0.858**	0.836**	-0.498*	0.602*	0.527*	0.266	0.388	-.763**	-0.435

* Indicates P < 0.05;

** Indicates P < 0.01.

Soil bulk density, BD; Soil organic matter, SOM; SM, Soil moisture; TN, Total nitrogen; TP, Total phosphorus; NN, Nitrate nitrogen; AN, Ammonium nitrogen; AP, Available phosphorus; AK, Available potassium; MBC, Soil microbial biomass carbon; C/N, The ratio of total organic carbon to total nitrogen; C/P, The ratio of total organic carbon to total phosphorus; N/P, The ratio of total nitrogen to total phosphorus.

**Fig 3:**
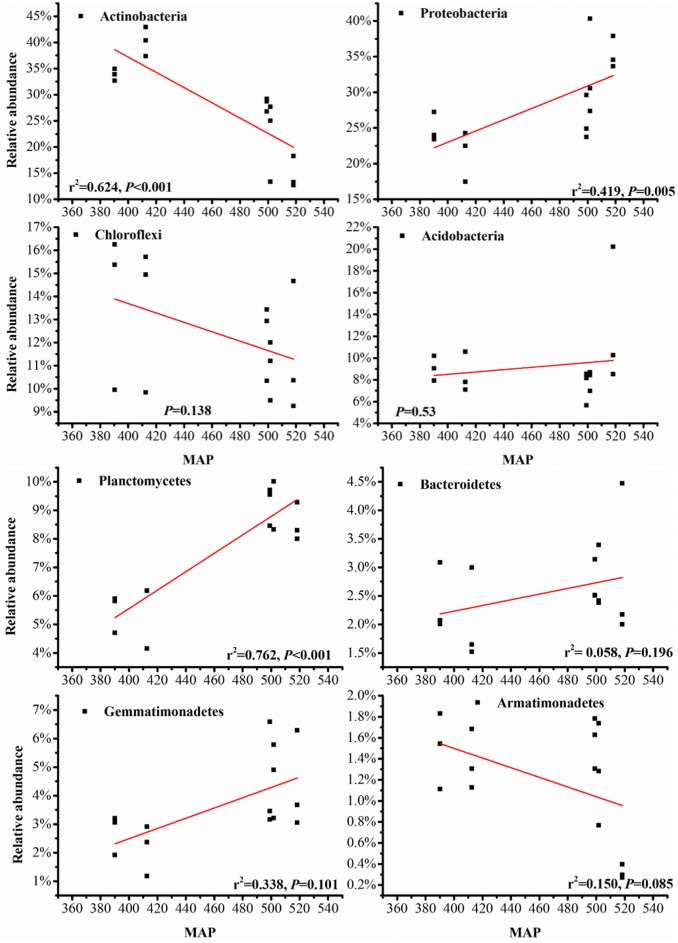
Ordination plots of the results from the RDA to examine the relationship between bacterial populations (shown in the blue) and selected environment factors (shown in red), including pH, TP, TN, MAP, MAT, MBC, SOM, SM and C/P in the five different ecosystems. Site 1, 2 and 3 were in the desert ecosystem; Site 4, 5 and 6 were in the sandy ecosystem; site 7, 8 and 9 were in the grass ecosystem; site 10, 11 and 12 were in the forest- grass ecosystem; site 13, 14 and 15 were in the forest ecosystem.

When the edaphic and climate variables were used to constrain the ordination of the soil bacterial (at the phylum level) with the RDA (Fig 3), there were distinct differences in bacterial community compositions under different ecosystems. We found that MAP, total P, SOM, TN, SM and C/P were the key factors that affected the soil bacterial compositions. The RDA analysis showed that there were clear soil bacteria community differences between sample sites, with desert and sandy ecosystems clusters differing from the grass and forest vegetation ecosystems (Fig 3). In desert and sandy ecosystems, *Actinobacteria* was the dominant phylum and affected by soil pH. In Grass and forest-grass ecosystems, *Proteobacteria* were the most abundant and the soil had the highest soil nutrients and MAP.

## Discussion

### Soil properties affecting soil bacteria compositions under different vegetation ecosystems

Soil chemical properties, including total C, total N, total P, and available N and K, decreased from south to north in the soils of the Loess Plateau in northwest China (Table 2). The changes in soil moisture demonstrated the same trend. However, soil pH and BD had the opposite trends, that is, they increased with increases in the MAP gradient. MAP was significantly associated with soil pH, BD, SM, SOM, TN, TP, NN, AN, AK, MBC, C/N, C/P and N/P, suggesting that the MAP diversity gradient was the key factor affecting the chemical and physical properties of the soil. This finding was similar to the biogeographic distribution of black soil properties from north to south in the northwest China [20].

Soil pH and water content also correlated with the bacterial community patterns. Although all of the samples were alkaline, the pH of the soils from the sandy and desert ecosystems was higher. The relative abundances of major bacterial classes in each soil type are consistent with predictions based on this difference in soil pH [21] in that there was a significant negative correlation of soil pH with *Proteobacteria* and a significant positive correlation with *Actinobacteria*. *Acidobacteria* were also sensitive to soil pH [22]. Our results showed that the strong influence of pH was also observed with respect to the major phyla as indicated by the observation that the relative abundances of *Actinobacteria*, *Proteobacteria* and *Armatimonadetes* across all sites changed significantly along the soil pH gradient (Table 4). Other environmental factors, such as temperature, might also be determinants of the soil bacterial community across this climate gradient in northwest China. Soil types also reflected differences in the biochemical composition of the litter, in terms of structural organic carbon and mineral nutrients, but the factors that are the best predictors of the composition and structure of soil bacterial communities remain to be identified [23]. In addition, microbial communities have been reported to respond to alterations in air temperature and humidity [24–25].

Sowerby et al. [26] observed a variation in the soil microbial extracellular enzymes in heath land ecosystems across geographical and climatic gradients from northern to southern Europe. With respect to the RDA, the soil moisture, SOM, TP, TN and C/P correlated significantly with soil bacterial communities (Fig 3). In this study, significant correlations were observed among of soil SOM, TN and TP. We therefore discuss only the relationship between the soil TP and composition of the bacterial communities in this paper. Soil microorganisms are the key regulators of the biogeochemical phosphorus cycle. Liu et al. [27] found that addition of P significantly increased the microbial biomass and altered the microbial community composition in old-growth forests, but there was no effect in pine forests. Cleveland et al. [28] found that the microbial activity was limited by P availability in tropical rain forests. Ehlers et al. [29] also observed a P limitation of the microbial growth after a combined addition of C and N to a native soil from western Kenya. Wang et al. [30] showed that P availability is vital to a priming effect in the subtropical forest soils and that P addition alone decreased soil respiration by 7.1%. In temperate forest ecosystems, the available pools of P strongly correlate with the microbial composition, and these authors found that from a microbial perspective soil, pH might be less important overall than P availability [31]. In our study, TP was significantly correlated with the relative abundance of *Actinobacteria*, *Proteobacteria*, *Planctomycetes* and *Gemmatimonadetes*. Thus, our results further emphasized that soil total P plays a key role in changing soil bacterial community compositions in the Loess Plateau.

Soil moisture was another of the most important factors that affected the bacterial communities. Soil moisture has strong effects on the soil properties and vegetation growth [32], consistent with our results. Soil moisture was the limiting factor for the microbial activities of 10 major phyla. Specifically, the relative abundances of *Actinobacteria*, *Proteobacteria*, *Acidobacteria*, *Planctomycetes* and *Armatimonadetes* were significantly correlated with soil moisture, especially for *Actinobacteria* and *Proteobacteria*. Soil moisture improves the microbes’ access to nutrients and also enhances their motility [33]. Soil moisture changed belowground C allocation. Drought reduced belowground C allocation and weakened the link between plant and bacterial. As a consequence, the more favorable soil moisture conditions in the soils could have allowed the microbes to maintain their activities, so soil moisture was a key factor for the soil microbes.

### Bacterial community composition in the soils of different vegetation ecosystems

Across all soil samples, the dominant groups (relative abundance >5%) in the Loess Plateau were *Actinobacteria*, *Proteobacteria*, *Chloroflexi*, *Acidobacteria* and *Planctomycetes*, consistent with previous studies on the soils of the Tibetan Plateau [34] and the black soils of northeast China [35]. Lauber et al. [36] found that *Acidobacteria*, *Actinobacteria*, *Proteobacteria* and *Bacteroidetes* are the dominant phyla in soils from North to South America [36], a result that is consistent with the observations of 29 soils from the Arctic region [37].

To the best of our knowledge, this study is the first to use a pyrosequencing approach to provide information concerning the bacterial diversity (richness and composition) in soils affected by various vegetation types after restoration in the Loess Plateau of China. Using this approach, the differences in the bacterial diversity among the five ecosystems were studied. Bacteria are the most abundant and diverse group of soil organisms [38–39]. Despite the key role of bacteria in soil processes, there is still a lack of information regarding the effects of restoration of vegetation ecosystems on bacterial diversity. We obtained OTUs to generate predictive rarefaction models and found differences in the richness (diversity) indices Ace and Chao1 as affected by the vegetation ecosystems evaluated. Generally, the soils of the grass ecosystem showed the highest diversity indices (Ace and Chao1) compared with other ecosystems.

Compared with the forest-grass, grass, sandy and desert ecosystems, the differences observed in bacterial diversity of the soils studied under the forest ecosystem is of ecological significance because this system takes more time to recover, and the soil has more nutrients and higher soil moisture. Due to the more diverse types of litter and rooting systems, the soils in the forest ecosystem have a higher biomass and soil total N, soil total P and soil total organic matter. In addition, our data are consistent with Chim et al. [39], who reported that the effect of the quality of soil organic materials associated with distinct vegetation types (as indicated by the C/N ratio) was stronger than their quantity effects on the structure of the soil bacterial community. The carbon and nutrient levels of the soils of different systems also varied considerably, and pronounced differences among the bacterial community structures among the vegetation types were observed.

In addition to fungal degradation of structural organic carbon, bacteria including Actinobacteria can solubilize complex carbon substrates such as lignin [40]. However, much less is known with respect to the degradation of structural organic carbon by *Acidobacteria* because few cultured representatives of this phylum are available for physiological studies. Recent *Acidobacterial* isolates from peat land soils are capable of degrading cellulose, albeit at a slow rate [41]. The plant litter quality among forest, shrub and grass ecosystems also differs markedly. Lignin is the main component of the organic carbon of tree wood debris, but grasses consist mostly of cellulose. In general, lignin has a higher C/N ratio than cellulose, which is consistent with the C/N ratios observed among the five vegetation systems on the Loess Plateau of China. Thus, forest, shrub and grass vary considerably in their substrate composition, as do the microbial communities that are involved in the degradation of the respective types of litter [42]. The roots of trees and grasses are additional major sources of organic matter to the soil, as well as sites of plant and soil microorganism interactions. Root exudates affect the soil bacterial community structure [43], and their quality and quantity may differ in different vegetation ecosystems. A correlation has been found between soil bacterial communities and carbon and nutrient contents, consistent with the findings obtained by Chan et al. [44]. In this study, the carbon and nitrogen levels of the forest ecosystem soils were the highest and could provide enough nutrients for the growth of bacteria.

### Bacterial community composition is affected by MAP

**Fig 4:**
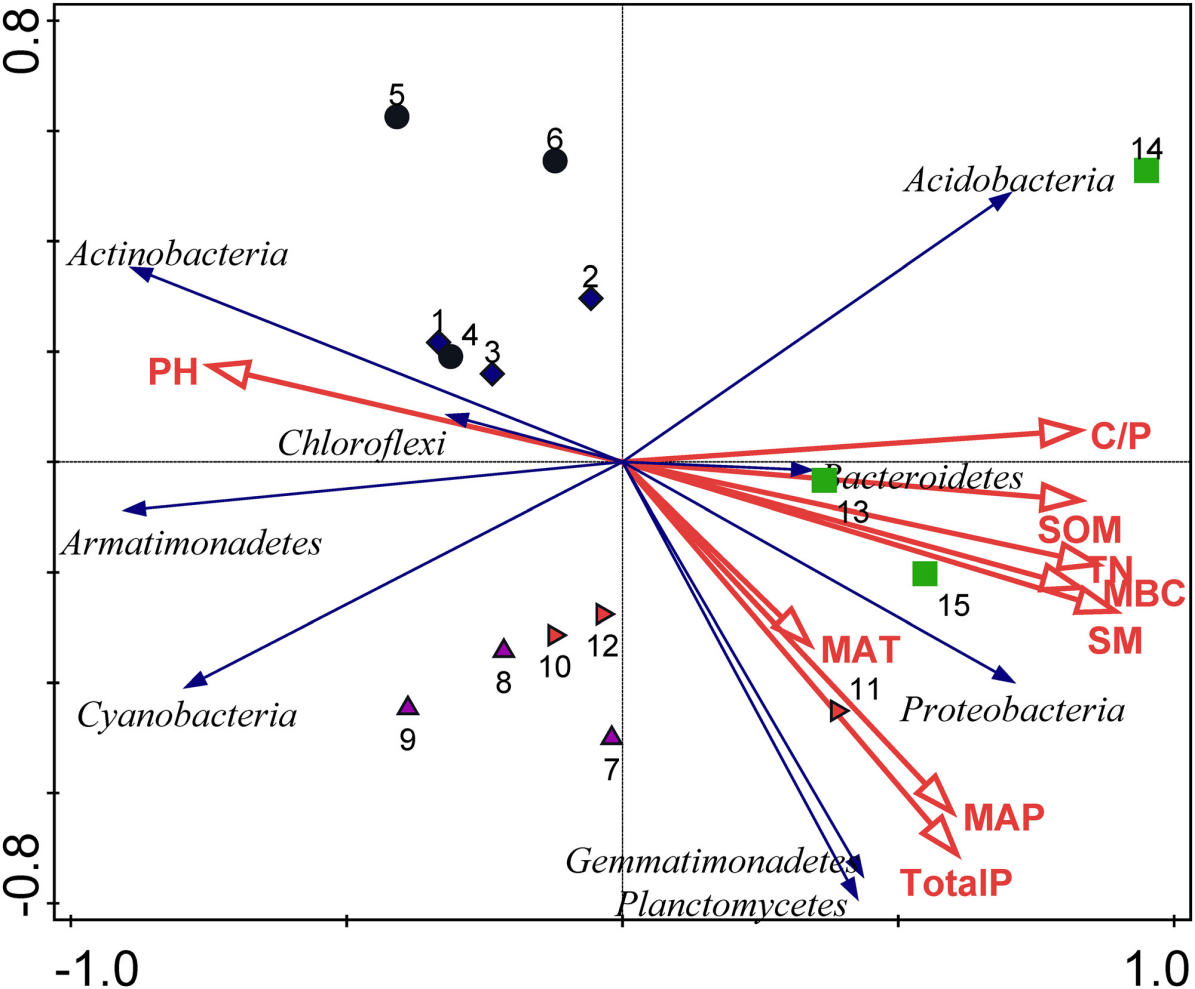
The relationship between latitude and the relative abundance within the soil bacterial community compositions (8 dominant phyla).

Many authors have reported that fungal diversity varies with the latitude gradient [45–47], and soil bacteria may show the same trend. In this study, we documented the overall pattern of the soil bacterial communities along an increasing latitudinal MAP from south to north on the Loess Plateau. Our results showed that soil properties had significant correlations with MAP, and soil bacterial community compositions correlated closely with the geographical location and the changes in MAP (Fig 4). The *Actinobacteria* phylum was poorly represented in the forest ecosystem with high MAP but predominated in the sandy ecosystem MAP. The *Proteobacteria* were the most abundant phylum in the forest ecosystem, and their abundance was lowest in the sandy ecosystem. Along the increasing MAP gradient, *Actinobacteria* and *Armatimonadetes* significantly increased, but *Proteobacteria*, and *Planctomycetes* significantly decreased. However, the relative abundances of *Chloroflexi*, *Acidobacteria*, *Bacteroidetes*, *Gemmatimonadetes* and Cyanobacteria were not significantly correlated with MAP (P>0.05). Hillebrand and Azovsky [48] hypothesized that the strength of the latitudinal gradient was positively correlated with body size. In contrast, Fuhrman et al. [49] reported that the diversity of the marine bacterial community decreased with increases in latitude. Fierer and Jackson [50] used a ribosomal DNA-fingerprinting method to investigate the relationship between the soil bacterial communities and conclude that edaphic variables (namely pH) mainly control bacterial biogeography. This is exactly in line with what this study finds. These results were consistent with those of Vries et al. [51]. The observed shift in life strategy between communities with a different precipitation history was accompanied by changes in the relative abundance of bacterial species which employed different strategies [52]. Microbial community composition and function are both sensitive to changes in rainfall [52]. Drought affects plants and soil microorganisms, especially for bacteria, not to fungi [53]. Variation in soil bacterial communities was explained by abiotic factors like climate (MAP), pH and soil properties.

## Conclusions

In this study, we analyzed the soil bacteria as a function of a MAP gradient on the Loess Plateau. Across all samples, the dominant groups were *Actinobacteria*, *Proteobacteria*, *Chloroflexi*, *Acidobacteria*, *Planctomycetes*, *Bacteroidetes*, *Gemmatimonadetes*, *Armatimonadetes* and Cyanobacteria. *Actinobacteria* and *Proteobacteria* dominated the five vegetation ecosystems (>14%) and were significantly correlated with soil moisture, C/P, MAP, and total P. Vegetation ecosystems had significant effect on soil bacteria composition. Ours is the first study to document the overall pattern of soil bacteria diversity along a MAP gradient on the Loess Plateau, and our results indicate that soil bacteria (*Actinobacteria*, *Proteobacteria* and *Planctomycetes*) follow the MAP diversity pattern of macroorganisms. Meanwhile, significant correlations between the relative abundance of soil bacteria (*Actinobacteria* and *Proteobacteria*) and pH were found in this study, suggesting that pH strongly influences soil bacterial communities. Furthermore, our data provide a starting point for establishing soil bacterial databases in the Loess Plateau, as well as for the plants associated with the vegetation restoration. These databases would provide a basis against which the impacts of future climate changes and ecosystem management practices can be assessed.

## Supporting Information

S1 Table Relevant data underlying the findings described in manuscript. (DOC)
