# Supplementary material for: Bacterial Community Responses to Soils along a Latitudinal and Vegetation Gradient on the Loess Plateau, China
Source: PLoS One. 2016 Apr 5;11(4):e0152894. doi: 10.1371/journal.pone.0152894 (PMC4821562; doi:10.1371/journal.pone.0152894)
Supplement: S1 Table — (DOC) [file pone.0152894.s001.doc]

S1 Table. Relevant data underlying the findings described in manuscript.

|  | Latitude | MAP | MAT | Altitude | pH | BD (g/cm^3^) | SM | SOM (g/kg) | TN (g/kg) | TP (g/kg) | NN (mg/kg) | AN (mg/kg) | AP (mg/kg) | AK (mg/kg) | MBC (mg/kg) | C/N | C/P | N/P |
| --- | --- | --- | --- | --- | --- | --- | --- | --- | --- | --- | --- | --- | --- | --- | --- | --- | --- | --- |
| 1 | 38.92 | 390.30 | 8.70 | 1192 | 8.35 | 1.72 | 0.01 | 3.24 | 0.10 | 0.19 | 0.99 | 4.43 | 2.38 | 178.90 | 19.80 | 18.86 | 10.13 | 0.54 |
| 2 | 38.82 | 390.30 | 8.70 | 1192 | 8.45 | 1.60 | 0.01 | 2.36 | 0.11 | 0.17 | 1.22 | 3.27 | 2.13 | 243.48 | 21.26 | 12.82 | 7.84 | 0.61 |
| 3 | 38.38 | 390.30 | 8.70 | 1120 | 8.27 | 1.66 | 0.02 | 5.26 | 0.18 | 0.29 | 2.31 | 2.55 | 2.76 | 207.59 | 26.86 | 16.52 | 10.55 | 0.64 |
| 4 | 37.65 | 412.60 | 9.40 | 1333 | 8.37 | 1.38 | 0.05 | 3.88 | 0.13 | 0.19 | 3.32 | 10.85 | 1.15 | 153.05 | 29.26 | 17.29 | 12.07 | 0.70 |
| 5 | 37.64 | 412.60 | 9.40 | 1349 | 8.24 | 1.63 | 0.01 | 1.32 | 0.06 | 0.11 | 3.30 | 4.32 | 1.35 | 186.73 | 15.32 | 13.19 | 6.79 | 0.51 |
| 6 | 37.86 | 412.60 | 9.40 | 1229 | 8.38 | 1.49 | 0.01 | 1.22 | 0.09 | 0.16 | 3.02 | 2.36 | 1.00 | 180.90 | 15.95 | 7.94 | 4.39 | 0.55 |
| 7 | 37.24 | 499.20 | 9.10 | 1463 | 8.22 | 1.23 | 0.08 | 8.96 | 0.57 | 0.48 | 6.66 | 4.81 | 1.44 | 312.42 | 83.02 | 9.10 | 10.75 | 1.18 |
| 8 | 37.18 | 499.20 | 9.10 | 1341 | 8.18 | 1.14 | 0.06 | 8.49 | 0.54 | 0.49 | 7.73 | 7.82 | 2.02 | 378.80 | 229.43 | 9.05 | 10.12 | 1.12 |
| 9 | 37.25 | 499.20 | 9.10 | 1406 | 8.26 | 1.21 | 0.04 | 7.78 | 0.42 | 0.45 | 4.00 | 5.40 | 1.82 | 284.06 | 171.37 | 10.63 | 9.96 | 0.94 |
| 10 | 36.75 | 501.80 | 10.30 | 1166 | 8.13 | 1.17 | 0.12 | 12.18 | 0.88 | 0.53 | 10.04 | 4.43 | 2.71 | 415.10 | 257.38 | 8.03 | 13.35 | 1.66 |
| 11 | 36.75 | 501.80 | 10.30 | 1153 | 8.07 | 1.20 | 0.18 | 23.98 | 1.87 | 0.56 | 12.30 | 10.95 | 2.82 | 259.13 | 457.11 | 7.44 | 25.05 | 3.37 |
| 12 | 36.86 | 501.80 | 10.30 | 1192 | 8.20 | 1.05 | 0.11 | 11.12 | 0.68 | 0.56 | 5.67 | 8.05 | 2.21 | 312.40 | 215.68 | 9.51 | 11.60 | 1.22 |
| 13 | 36.06 | 518.30 | 9.50 | 1333 | 7.84 | 0.97 | 0.15 | 49.89 | 2.55 | 0.62 | 1.58 | 11.54 | 2.30 | 508.27 | 612.18 | 11.36 | 46.93 | 4.13 |
| 14 | 36.06 | 518.30 | 9.50 | 1327 | 7.86 | 0.69 | 0.24 | 44.77 | 2.06 | 0.52 | 5.74 | 10.24 | 2.08 | 557.58 | 693.15 | 12.63 | 50.14 | 3.97 |
| 15 | 36.08 | 518.30 | 9.50 | 1152 | 8.14 | 1.16 | 0.15 | 18.34 | 1.13 | 0.57 | 8.01 | 6.21 | 2.43 | 525.52 | 350.83 | 9.42 | 18.67 | 1.98 |

^a^Soil bulk density, BD; Soil organic matter, SOM; SM, Soil moisture; TN, Total nitrogen; TP, Total phosphorus; NN, Nitrate nitrogen; AN, Ammonium nitrogen; AP, Available phosphorus; AK, Available potassium; MBC, Soil microbial biomass carbon; C/N, The ratio of total organic carbon to total nitrogen; C/P, The ratio of total organic carbon to total phosphorus; N/P, The ratio of total nitrogen to total phosphorus.
